# TFCONES: A database of vertebrate transcription factor-encoding genes and their associated conserved noncoding elements

**DOI:** 10.1186/1471-2164-8-441

**Published:** 2007-11-29

**Authors:** Alison P Lee, Yuchen Yang, Sydney Brenner, Byrappa Venkatesh

**Affiliations:** 1 Institute of Molecular and Cell Biology, 61 Biopolis Drive, Singapore 138673, Singapore

## Abstract

**Background:**

Transcription factors (TFs) regulate gene transcription and play pivotal roles in various biological processes such as development, cell cycle progression, cell differentiation and tumor suppression. Identifying *cis*-regulatory elements associated with TF-encoding genes is a crucial step in understanding gene regulatory networks. To this end, we have used a comparative genomics approach to identify putative *cis*-regulatory elements associated with TF-encoding genes in vertebrates.

**Description:**

We have created a database named TFCONES (Transcription Factor Genes & Associated COnserved Noncoding ElementS) () which contains all human, mouse and fugu TF-encoding genes and conserved noncoding elements (CNEs) associated with them. The CNEs were identified by gene-by-gene alignments of orthologous TF-encoding gene loci using MLAGAN. We also predicted putative transcription factor binding sites within the CNEs. A significant proportion of human-fugu CNEs contain experimentally defined binding sites for transcriptional activators and repressors, indicating that a majority of the CNEs may function as transcriptional regulatory elements. The TF-encoding genes that are involved in nervous system development are generally enriched for human-fugu CNEs. Users can retrieve TF-encoding genes and their associated CNEs by conducting a keyword search or by selecting a family of DNA-binding proteins.

**Conclusion:**

The conserved noncoding elements identified in TFCONES represent a catalog of highly prioritized putative *cis*-regulatory elements of TF-encoding genes and are candidates for functional assay.

## Background

Transcription factors (TFs) are proteins that bind to *cis*-regulatory elements and activate or repress transcription of genes. Other proteins that are involved in transcriptional regulation and that could fall under the broad classification of TFs, include co-factors, chromatin remodeling enzymes involved in chromatin or histone modification affecting the transcriptional state of target genes, and general transcription factors that associate directly with RNA polymerase II in a transcription initiation complex. The human genome is estimated to contain about 2,000 TF-encoding genes including co-factors, chromatin remodeling enzymes and general transcription factors [1]. TFs play crucial roles in development (e.g., HOX, SOX and PAX proteins), cell cycle progression (e.g., c-MYC, c-JUN), tumor suppression (e.g., p53, FOXO proteins) and cell differentiation (e.g., RUNX, DLX proteins). The vast majority of TFs are known to regulate the expression of a number of different genes, and TF-encoding genes themselves are the key targets of TFs, with many TFs regulating the expression levels of their own genes. Thus, TFs represent crucial nodes in the gene regulatory networks that determine the correct development of the body plan and regulate various physiological processes. To gain an understanding of these gene regulatory networks, it is important to identify *cis*-regulatory elements associated with TF-encoding genes and the TFs involved in the differential expression of TF-encoding genes. Mutations that disrupt the *cis*-regulatory elements of TF-encoding genes have been implicated in genetic diseases such as aniridia (disruption of *PAX6 *regulatory elements, [2]), Rieger syndrome (*PITX2 *[3,4]) and campomelic dysplasia (*SOX9 *[5]).

With the availability of whole genome sequences of several vertebrates, comparative genomics has proved to be a powerful approach for identifying *cis*-regulatory elements in vertebrate genomes. Since functional noncoding elements tend to evolve slowly due to selective pressure, potential *cis*-regulatory elements can be identified as noncoding sequences that are conserved in distantly related genomes. Whole-genome comparisons of human and other vertebrates have indeed proved to be effective in identifying functional *cis*-regulatory elements in the human genome [6-11]. Notably, a large number of conserved noncoding elements (CNEs) identified in these genome-wide comparisons were found to be associated with TF-encoding and developmental genes. For example, 83% (1,140 out of 1,373) of CNEs (>70% identity/>100 bp alignment length) identified in genome-wide comparison of human and fugu were located in the vicinity of about 120 human DNA-binding TF-encoding genes [10], while 104 of the 290 human genes associated with human-zebrafish CNEs (>70% identity/>80 bp sequence length) were found to be TF-encoding genes [9]. In spite of such a known association between TF-encoding genes and CNEs, no systematic gene-by-gene comparison of all the orthologous human and other vertebrate TF-encoding genes has been carried out to identify potential *cis*-regulatory elements associated with them. Whole-genome comparisons, particularly between distantly related genomes such as human and fish, fail to identify and align all the orthologous sequences due to the stringent criterion of local alignment algorithms. On the other hand, locus-by-locus comparison of orthologous TF-encoding genes is more effective in identifying all the associated CNEs. Furthermore, because global alignment algorithms have an additional assumption that input sequences occur in the same order and orientation, they have more power in detecting weakly conserved regions than local alignments [12,13]. Nevertheless, it should be noted that global alignment algorithms tend to miss conserved functional elements that have undergone local inversions and rearrangements. Previously, orthologous TF-encoding genes of human, rodents, fugu and zebrafish have been compared [14-17]. However, such studies were promoter-centric, with comparisons restricted to sequences flanking the transcription start sites. The scope of such studies is limited because *cis*-regulatory elements can be located at considerable distance from transcription start sites, even up to several hundred kilobases away [18]. More recently, a selected set of developmentally regulated genes and their associated conserved noncoding elements has been reported [19]. This database includes many but not all of the TF-encoding genes.

We have constructed a database of DNA-binding transcription factor-encoding genes in vertebrate genomes and evolutionarily conserved noncoding elements associated with them. This database would be useful to researchers interested in studying the regulation of TF-encoding genes and understanding gene regulatory networks in vertebrates.

## Construction and Content

### Human, mouse and fugu transcription factor genes

Sequences for 1,962 human TFs were obtained from [1] and redundancies were removed by a homology search against human RefSeq proteins. Several known proteins missing from Messina et al.'s dataset (e.g., *JMJ2A, JMJ2C, JMJ2D, HES4 *and *DLX6*) were included with the search results and the resulting proteins were mapped to human genes in Ensembl Release 37 [20]. The TFs were classified by DNA-binding domains or if lacking a DNA-binding domain, the TFs were classified separately into one of the following categories: co-factors, general transcription factors, components of chromatin remodeling complexes and transcriptional regulators that are involved solely in protein-protein interactions (i.e., TFs with ZnF-PHD, ZnF-BTB/POZ, ZnF-MYND domains). Mouse orthologs were retrieved from Ensembl BioMart. Fugu orthologs were identified using a combination of data from Ensembl BioMart (fugu version 4 assembly) and INPARANOID analysis [21]. Fishes contain duplicate copies for many human genes due to a 'fish-specific' whole-genome duplication event in the fish lineage [22]. INPARANOID was used to identify duplicate fugu orthologs for human TFs that may have been missed in Ensembl. Only proteins longer than 50 residues were used in the analysis. INPARANOID identified some many-to-many ortholog groups which were resolved into smaller families based on phylogenetic analysis using PHYLIP [23] with sequences from cartilaginous fishes, lamprey, amphioxus or *Ciona intestinalis *as the outgroup. For each family, multiple human and fugu proteins and a single outgroup sequence were aligned with ClustalW. Alignments with ungapped alignment length less than 50 residues were rejected and consensus trees were generated for the remaining alignments using 1,000 bootstraps and the PHYLIP programs PROTDIST and NEIGHBOR (Neighbor-Joining method). Following the generation of phylogenetic trees, families were resolved to one-to-one or one-to-two human-to-fugu gene relationships based on visual inspection of the consensus trees. TF-encoding genes present in clusters (linked to each other on a chromosome/scaffold, with no intervening non-TF-encoding gene) in the genome were identified using a pre-computed index of the relative locations of all protein-coding genes in Ensembl Release 37. To avoid redundancy in predicting conserved noncoding elements associated with TF-encoding genes that reside in clusters, we identified conserved clusters of human TF-encoding genes whose orthologs in mouse and fugu are conserved in the same order and orientation.

### Aligning human-mouse-fugu TF-encoding gene loci and identifying CNEs

**Figure 1:**
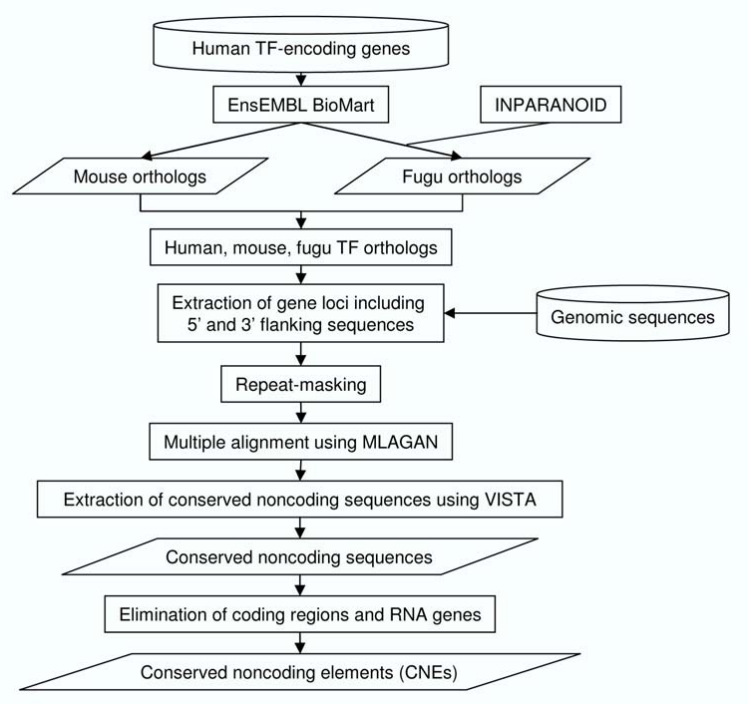
A flowchart of protocol used for identifying human-mouse and human-fugu conserved noncoding elements.

The protocol used for identifying CNEs associated with human TF-encoding genes is summarized in Fig 1. Genomic sequences of human, mouse and fugu TF-encoding gene loci (spanning entire conserved clusters or singleton genes) including the entire 5' and 3' flanking regions (up to the next gene upstream and downstream), were retrieved from Ensembl Release 37. The sequences were repeat-masked using RepeatMasker version open-3.1.5 [24]. Sequences of human, mouse and fugu orthologous gene loci were aligned using MLAGAN [25]. Multiple alignments between human, mouse and fugu sequences were preferred over pair-wise alignments between human and fugu sequences, because multiple alignments are generally more selective (fewer false positives) than pair-wise alignments in the detection of regulatory elements [26]. A global alignment strategy is suitable for our goal of aligning orthologous TF-encoding gene loci which are assumed to occur in the same order and orientation. However, it should be noted that global alignment algorithms are unable to detect conserved functional elements that have undergone local inversions and rearrangements.

Conserved noncoding sequences were visualized and detected with VISTA [27] using the fugu sequence as the reference sequence. Conserved noncoding sequences between human and mouse were defined as regions with minimum 70% identity over 100 bp of sequence [28]. Considering the longer evolutionary distance between human and fugu, and the fact that BLASTZ alignments of human and fugu genomes show that the alignable regions cover only 1.8% of the human genome with an average identity of 60% [6], we defined conserved noncoding sequences between mammals and fugu as sequences that display minimum 65% identity over 50 bp of sequence. Any conserved noncoding sequences that overlapped with protein-coding sequence (Ensembl Release 37 or BLASTX search against NCBI's non-redundant proteome, E-value < 10^-4^) were eliminated. Pseudogenes and noncoding RNA genes annotated in Ensembl Release 37 were also eliminated. Conserved noncoding sequences that contained RNA genes were identified based on BLASTN (E-value < 10^-4^) and INFERNAL searches against Rfam (Release 7.0) and miRBase (Release 8.0), and excluded from further analysis. Sequences containing 20% or more microsatellite repeats (2 to 5 nucleotides with minimum 4 occurrences and minimum 10 bp in length) were also filtered out. The remaining conserved noncoding sequences were reclassified as conserved noncoding elements (CNEs). CNEs were annotated as " 5' ", "intronic" or " 3' " based on their location with respect to the protein-coding sequence of human TF-encoding genes. CNEs that fall within intergenic regions of TF-encoding genes are annotated with respect to both upstream and downstream genes.

### Analysis of CNE statistics for human TF-encoding genes

For every orthologous human TF-encoding gene, we determined the density, number and total length of human-fugu CNEs associated with that gene. CNE density is defined as the number of bases residing in CNEs per unit length (1 kilobase in human; 100 bp in fugu) of non-repetitive noncoding sequence in a human gene locus or the longest orthologous fugu gene locus. For genes that are part of conserved clusters, we averaged out the number or length of CNEs present in the whole cluster over the number of genes in that cluster. For CNE density, we averaged the total length of CNEs present in the whole cluster over the non-repetitive noncoding sequence length of the cluster.

### Gene Ontology enrichment analysis

To test the null hypothesis that human TF-encoding genes containing human-fugu CNEs are independent of their associated Gene Ontology (GO) terms, a Fisher's exact test was applied in the GOstat package [29]. Gene Ontology terms of genes that contained CNEs were submitted to GOstat for analysis against all the human TF orthologs. Two-tailed *P*-values were reported. Only the "biological process" subset of GO hierarchy was considered. GO output was restricted to GO-terms with *P*-value < 0.01, and the "false discovery rate" method [30] was used for multiple testing correction. The reference GO annotation was retrieved from Ensembl BioMart for all human protein-coding genes.

### Enrichment analysis of CNEs within experimentally defined TF binding sites

The human-fugu CNEs that overlap with the binding sites of OCT4, SOX2, NANOG [31], c-MYC [32] and SUZ12 [33] were identified, by comparing chromosomal coordinates of CNEs and TF bound regions and counting CNEs with at least half of its length overlapped by a bound region. Except the bound regions of c-MYC which were identified by the ChIP-PET technique, all other bound regions were identified by ChIP-on-chip.

To test the null hypothesis that the number of CNEs that overlap experimentally validated TF binding sites is no greater than that expected from CNEs that overlap randomized TF binding sites, a binomial distribution was applied in the following way. For every bound region identified by ChIP-on-chip, we generated a random genomic region of the same length by randomly choosing a probe present on the microarray chip and centering the random genomic region on the chosen probe. For c-MYC, random regions were sampled from the entire human genome. After each randomization, the proportion of CNEs that overlapped the random regions was determined. The random process was repeated 1000 times and a binomial distribution was applied using the expectation derived from the overlap of CNEs with random regions. One-tailed *P*-values were calculated.

### Prediction of TF binding sites

TF binding sites were detected in human-fugu CNEs using TESS (TRANSFAC 4.0 data) [34]. Only vertebrate sites and weight matrices were searched with background (A+T)-content of 60.4% (average (A+T)-content for all human CNE sequences) or 57.4% (average (A+T)-content for all fugu CNE sequences). Overlapping binding sites bound by the same TFs were removed, and only string matches with log likelihood score ratio *L*_*q *_> 0.98 and matrix matches with core similarity *S*_*c *_> 90% and matrix similarity *S*_*m *_> 80% were retained.

### Implementation of TFCONES database

The TFCONES database is housed in a MySQL server and made accessible via an Apache web server. Perl scripts are used to generate result pages in response to user queries. The Generic Genome Browser [35] is used for the display of CNEs on the human genome. A local *wwwblast *server [36] has been set up to allow users to BLAST-search their sequences against the CNEs in the TFCONES database. In order to provide a set of high-confidence TF binding sites located in CNEs that would be useful for experiments, only human TF binding sites that overlap an orthologous prediction in the fugu sequence are displayed in the database.

## Utility and Discussion

### Transcription factor-encoding genes in human, mouse and fugu

In the TFCONES database, we have identified in total 1,738 human, 1,495 mouse and 1,762 fugu TF-encoding genes. For the purpose of identifying conserved *cis*-regulatory elements associated with a set of TF-encoding genes that regulate transcription in the same way (i.e., via DNA-binding), we excluded the co-factors, chromatin modifiers and general transcription factors from further analysis and focused on DNA-binding transcription factors. This resulted in the identification of 1,327 sequence-dependent or DNA-binding transcription factors. Similarly, 1,086 and 1,328 DNA-binding TFs were identified in mouse and fugu respectively. Almost all families of TFs, except C_2_H_2_-type (cysteine_2_-histidine_2_) zinc finger proteins, contain a higher number of genes in fugu than human and mouse (Table 1). These additional TFs in fugu are likely to be the result of the 'fish-specific' whole-genome duplication in the fish lineage [22]. We identified the mouse and fugu orthologs for human TF-encoding genes using reciprocal BLAST and INPARANOID (see Construction and Content). Of the 1,327 human genes that encode DNA-binding transcription factors, 1,069 genes have orthologs in mouse while 816 genes have orthologs in both mouse and fugu. Fugu contains two or more orthologs for 36 human TF-encoding genes (Additional data file 1).

**Table 1:** DNA-binding TF-encoding genes in human, mouse and fugu genomes. The TFs were classified based on their DNA-binding domains using a classification scheme adapted from Messina et al. [1]

**TF family**	**Human**	**Mouse**	**Fugu**
AP-2	5	5	6
ARID	11	9	15
Beta-scaffold – CCAAT	8	8	9
Beta-scaffold – MADS	5	5	9
Beta-scaffold – p53	3	3	3
Beta-scaffold – RUNT	3	3	5
Beta-scaffold – Others	28	28	31
BHLH	96	94	134
BZIP	69	65	83
Dwarfin	8	8	13
E2F	11	9	11
Forkhead	40	35	60
GCM	2	2	2
Heat shock factor	7	4	10
High mobility group box	35	39	56
Homeobox	221	199	282
Nuclear hormone receptor	49	49	69
Paired box	9	9	14
RFX	6	6	7
T-box	16	15	19
TEA	4	4	5
Trp cluster – Ets	29	29	31
Trp cluster – IRF	9	9	15
Trp cluster – Myb	11	11	13
ZnF-C2H2	472	290	254
ZnF-C3H	6	4	7
ZnF-DM	9	7	6
ZnF-GATA	10	10	12
ZnF-Others	61	55	73
Others (e.g., SAND, RBPSUH)	84	72	74

**Total**	**1,327**	**1,086**	**1,328**

### Conserved clusters of TF-encoding genes in human, mouse and fugu

TF-encoding genes such as *Hox*, *Iroquois *and *Dlx *genes exist in clusters. The conservation of clustered organization of genes in distantly related genomes is thought to be related to the presence of evolutionarily constrained shared enhancers [37-39]. We identified a total of 149 clusters of human TF-encoding genes containing 407 genes, 95 clusters of mouse TF-encoding genes containing 253 genes, and 115 clusters of fugu TF-encoding genes containing 280 genes (Additional data files 2, 3 and 4). Of these clusters, 18 orthologous clusters comprising 70 TF-encoding genes were found to be conserved in order and orientation in all three genomes (Additional data file 5). These evolutionarily conserved TF-encoding gene clusters are likely to be associated with evolutionarily constrained shared enhancers. This set includes instances such as HoxD cluster which has been shown to contain conserved enhancers that regulate several genes in the cluster [37]. Among these clusters, the identification of CNEs in the four human *Hox *gene clusters has been previously reported [40].

### Identification of human-mouse and human-fugu CNEs

After alignment of the orthologous human, mouse and fugu TF-encoding gene loci using MLAGAN [25] and detection of CNEs using VISTA [27] and subsequent filtering steps, of the 816 human-mouse-fugu orthologous TF-encoding genes, 797 genes contain 58,954 human-mouse CNEs, whereas 389 genes contain 2,843 human-fugu CNEs. The human-mouse and human-fugu CNEs represent 5.7% and 0.15% respectively, of noncoding sequences extracted for alignments. The distribution of the lengths of human-mouse and human-fugu CNEs are presented in Additional data file 6. The human-mouse CNEs are on average 244 bp long (median is 183 bp; longest is 3.9 kb) and add up to a total length of 14,375 kb. The average length of human-fugu CNEs is 136 bp (median is 106 bp; longest is 1.0 kb) and the total length is 388 kb. Whole-genome comparison of human and mouse has indicated that about 5% of human and mouse sequences are under evolutionary constraint [41], of which 3.5% are noncoding sequences. The "Gumby" algorithm developed by Prabhakar et al. [42] which is based on human-rodent sequence comparisons and enforces significance thresholds, predicts that only 2.2% of noncoding sequences in the human and rodent genomes are likely to be under selection. It is, therefore, likely that a considerable number of the human-mouse CNEs are not under evolutionary constraint but rather sequences that did not have adequate time to diverge. On the other hand, most of the human-fugu CNEs which are conserved over a long evolutionary period (420 Myr) are likely to be under purifying selection. To verify this, we determined if the human-fugu CNEs significantly overlap with experimentally validated transcription factor binding sites. We also computed the density and distribution of human-fugu CNEs among TF-encoding genes. The results of these analyses are presented below.

### Density and number of human-fugu CNEs per gene

**Figure 2:**
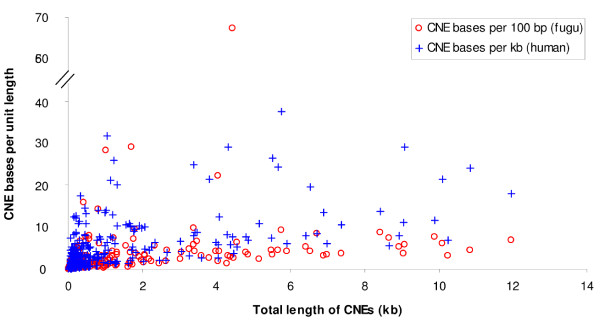
Plot of CNE density against the total length of CNEs associated with DNA-binding TF-encoding genes. CNE density is defined as the number of bases located in CNEs per unit length (1 kb in human; 100 bp in fugu) of non-repetitive noncoding sequence in a gene locus.

Since we used the entire 5' and 3' flanking regions in our alignments, we investigated whether genes with larger flanking regions were associated with more human-fugu CNEs than genes with short flanking regions. To this end, we calculated the density of human-fugu CNEs in human TF-encoding genes. The CNE densities of human genes vary widely from 0.03 to 38 bp per kb of human sequence for the 389 CNE-associated genes (Fig 2). The top 20 human TF-encoding genes with the highest CNE densities are listed in Table 2. These genes are characteristically homeodomain-encoding genes such as the genes XP_496843 (*Uncx4.1*), XP_291716 (*HMX3*), NP_005510 (*HMX2*), *LMO1 *and *PBX3*. The high CNE densities of these genes indicate that a large proportion of their noncoding regions are under evolutionary constraint. To determine if the compaction of intergenic regions in fugu has affected CNE density, we also measured CNE densities in fugu for every human gene, which we defined as the number of bases residing in CNEs per 100 bp of the longest orthologous fugu gene locus. The CNE density in fugu also ranges widely from 0.02 to 67 bp per 100 bp of fugu sequence (Fig 2). However, 16 of the top 20 genes (human and their fugu orthologs) with the highest human-fugu CNE densities in fugu (Additional data file 7) are different from the top 20 genes of highest CNE densities in human (Table 2). This indicates that the intergenic regions have not been compacted or expanded uniformly in fugu and human, respectively.

**Table 2:** Top twenty TF-encoding genes associated with the highest density of human-fugu CNEs. For genes that are part of conserved clusters, we averaged out the number or length of CNEs present in the whole cluster over the number of genes in that cluster. CNE density is defined here as the number of bases located in CNEs per kilobase of non-repetitive noncoding sequence in a gene locus.

**Gene ID**	**Gene name**	**Description**	**CNE bases per kb of human sequence**
ENSG00000164853	*XP_496843.1*	PREDICTED: similar to Uncx4.1	37.61
ENSG00000188620	*XP_291716.5*	PREDICTED: similar to homeodomain protein	31.82
ENSG00000188816	*NP_005510.1*	Homeobox (H6 family) 2	31.82
ENSG00000166407	*LMO1*	Rhombotin-1	29.17
ENSG00000167081	*PBX3*	Pre-B-cell leukemia transcription factor 3	29.16
ENSG00000130940	*CASZ1*	Probable transcription factor CST	26.43
ENSG00000139800	*ZIC5*	Zinc finger protein ZIC 5	25.94
ENSG00000109132	*PHOX2B*	Paired mesoderm homeobox protein 2B	24.93
ENSG00000075891	*PAX2*	Paired box protein Pax-2.	24.27
ENSG00000177508	*IRX3*	Iroquois-class homeodomain protein IRX-3	24.04
ENSG00000165655	*ZNF503*	zinc finger protein 503	21.39
ENSG00000143032	*BARHL2*	BarH-like 2 homeobox protein.	21.28
ENSG00000171540	*OTP*	orthopedia	21.25
ENSG00000125285	*SOX21*	Transcription factor SOX-21	19.99
ENSG00000159387	*IRX6*	Iroquois-class homeodomain protein IRX-6	19.51
ENSG00000176842	*IRX5*	Iroquois-class homeodomain protein IRX-5	19.51
ENSG00000134138	*MEIS2*	Homeobox protein Meis2	18.05
ENSG00000128652	*HOXD3*	Homeobox protein Hox-D3	17.34
ENSG00000128709	*HOXD9*	Homeobox protein Hox-D9	17.34
ENSG00000128710	*HOXD10*	Homeobox protein Hox-D10	17.34

**Figure 3:**
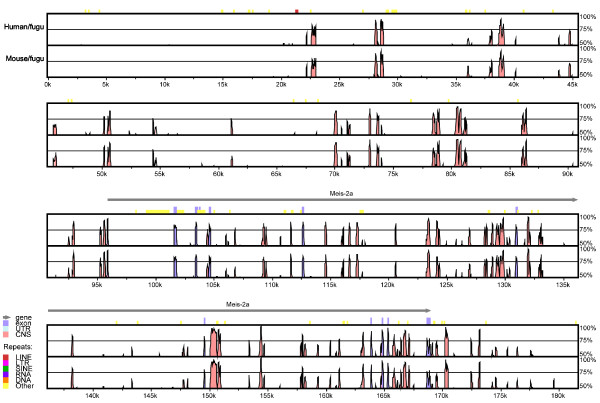
Human-fugu and mouse-fugu VISTA alignments of *MEIS2 *gene locus. Pink peaks denote conserved noncoding elements (CNEs) and blue peaks denote conserved exonic sequences. *MEIS2 *locus contains the highest number of CNEs (79 CNEs with a total length of 12.0 kb) among TF-encoding genes in the human, mouse and fugu genomes.

The number of CNEs associated with each gene gives an indication of the number of conserved *cis*-regulatory elements in each of these genes. The top 20 human genes with the highest number and total length of human-fugu CNEs are shown in Table 3. The *MEIS2 *locus contains the highest number and total length of CNEs (79 CNEs spanning 12.0 kb) (Fig 3). In mammals, *Meis2 *is involved in the differentiation of the forebrain, branchial arches and somitic mesoderm as well as in regulating limb outgrowth [43,44]. The next gene with the most CNEs is *IRX3 *(68 CNEs spanning 10.9 kb). *IRX3 *is expressed in certain regions of the midbrain, hindbrain, otic vesicle, spinal cord, first and second branchial arches and proximodorsal regions of the developing limb buds [45]. The association of an abundance of CNEs with TF-encoding genes such as *MEIS2 *and *IRX3 *that show spatially restricted expression patterns in a wide range of tissues is consistent with the notion that these CNEs represent regulatory modules that direct expression to different expression domains. At the other end of the spectrum are the TF-encoding genes that do not contain any human-fugu CNEs. TF-encoding genes that lack CNEs and exhibit ubiquitous expression (e.g., *SP1, USF1, E2F1*) may contain strong basal promoters and lack tissue-specific enhancers. In the case of CNE-lacking TF-encoding genes that exhibit tissue-specific expression, the divergent regulatory elements in human and fugu may still confer the same expression patterns in human and fugu as demonstrated in the case of human and zebrafish RET gene regulatory elements [46]. It is also possible that some of these divergent regulatory elements may have conferred different patterns of expression in human and fugu.

**Table 3:** Top twenty TF-encoding genes associated with the highest number and total length of human-fugu CNEs. For genes that are part of conserved clusters, we averaged out the number or length of CNEs present in the whole cluster over the number of genes in that cluster.

**Gene ID**	**Gene name**	**Description**	**Number of CNEs**	**Length of CNEs (kb)**
ENSG00000134138	*MEIS2*	Homeobox protein Meis2	79	11.98
ENSG00000177508	*IRX3*	Iroquois-class homeodomain protein IRX-3	68	10.87
ENSG00000169554	*ZFHX1B*	Zinc finger homeobox protein 1b	76	10.25
ENSG00000143032	*BARHL2*	BarH-like 2 homeobox protein.	56	10.09
ENSG00000169946	*ZFPM2*	Zinc finger protein ZFPM2	49	9.90
ENSG00000167081	*PBX3*	Pre-B-cell leukemia transcription factor 3	57	9.10
ENSG00000091656	*ZFHX4*	zinc finger homeodomain 4	77	9.05
ENSG00000151514	*SALL3*	Sal-like protein 3	59	8.94
ENSG00000170549	*IRX1*	Iroquois-class homeodomain protein IRX-1	48	8.67
ENSG00000128573	*FOXP2*	Forkhead box protein P2	57	8.41
ENSG00000121297	*ZNF537*	Teashirt homolog 3	59	7.38
ENSG00000143995	*MEIS1*	Homeobox protein Meis1.	63	7.00
ENSG00000148737	*TCF7L2*	Transcription factor 7-like 2	42	6.91
ENSG00000153234	*NR4A2*	Orphan nuclear receptor NR4A2	38	6.73
ENSG00000159387	*IRX6*	Iroquois-class homeodomain protein IRX-6	40.5	6.54
ENSG00000176842	*IRX5*	Iroquois-class homeodomain protein IRX-5	40.5	6.54
ENSG00000110693	*SOX6*	Transcription factor SOX-6.	47	6.41
ENSG00000143013	*LMO4*	LIM domain transcription factor LMO4	32	5.92
ENSG00000164853	*XP_496843.1*	PREDICTED: similar to Uncx4.1	31	5.74
ENSG00000075891	*PAX2*	Paired box protein Pax-2.	37	5.68

### Human-fugu CNEs overlap experimentally verified TF binding sites indicating they contain transcriptional regulatory elements

The recent advances in the high-throughput techniques for identifying *in vivo *binding sites of TFs such as ChIP-on-chip/ChIP-PET have enabled genome-wide mapping of binding sites for TFs such as OCT4, SOX2, NANOG, c-MYC and SUZ12 [31-33]. Although genome-wide ChIP analysis has a certain level of noise and does not give conclusive evidence of transcriptional regulation, it provides a good estimate of putative regulatory elements. If the human-fugu CNEs identified by us represent transcriptional regulatory elements, a significant proportion of them should overlap with experimentally verified TF binding sites. To verify this we searched the human-fugu CNEs against the human genome sequences that were shown to be bound by various TFs ("bound regions").

The TFs OCT4, SOX2 and NANOG are crucial for the maintenance of pluripotency and self-renewal in embryonic stem (ES) cells and are necessary for propagation of undifferentiated ES cells in culture [31]. These factors activate the transcription of their own genes and genes of major signaling pathways, but repress the transcription of some developmental genes [31]. Sequences bound by these TFs within promoter regions of genes in human ES cells were taken from Boyer et al. [31]. A significant proportion of human-fugu CNEs were found to overlap bound regions of OCT4 (0.63% of human-fugu CNEs; *P *= 1.82 × 10^-17^), SOX2 (0.84% of human-fugu CNEs; *P *= 8.83 × 10^-18^) and NANOG (1.27% of human-fugu CNEs; *P *= 3.44 × 10^-26^). The transcription factor c-MYC plays important roles in regulating cell growth, cell proliferation, cell cycle and apoptosis [47]. The genomic bound regions of c-MYC in human B cells were recently identified using ChIP-PET experiments [32]. In total, about 4,300 high-confidence bound regions of c-MYC were identified to be associated with 668 target genes, of which 48 encode TFs [32]. We found that 0.49% of the human-fugu CNEs overlap c-MYC bound regions, constituting a significant enrichment of c-MYC binding sites in CNEs (*P *= 4.44 × 10^-5^). SUZ12 is a subunit of the polycomb repressive complex 2 which is involved in silencing of genes through the epigenetic modification of chromatin structure [33]. A significant proportion of human-fugu CNEs (10.45%; *P *= 1.53 × 10^-316^) were found to overlap SUZ12 bound regions in human ES cells [33]. The significant association of human-fugu CNEs with experimentally verified TF binding sites of both transcriptional activators and repressors, indicates that a significant proportion of CNEs are functional transcriptional enhancers and silencers.

### TF-encoding genes associated with CNEs tend to be involved in nervous system development

To determine the relationship between human-fugu CNEs and biological functions of TF-encoding genes, the Gene Ontology (GO) terms describing the biological processes of CNE-associated TF-encoding genes (385 genes with Gene Ontology annotation) were compared with that of all human TF orthologs (804 genes with Gene Ontology annotation) using GOstat [29]. TF-encoding genes associated with CNEs were found to be significantly enriched for genes involved mainly in development, and in particular development of the nervous system (*P *< 0.01) (Additional data file 8). In contrast, TF-encoding genes that are associated with CNEs are depleted of genes involved in protein metabolism and biopolymer modification (*P *< 0.01) (Additional data file 8; *P*-values marked with a negative sign). The significant involvement of CNE-associated TF-encoding genes in nervous system development is consistent with previous studies that highlight the association of highly conserved noncoding regions with developmental genes [6,8,10].

### Potential utility of the TFCONES database

**Figure 4:**
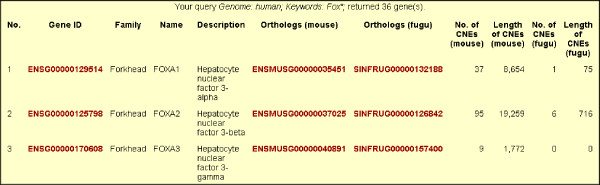
A keyword search of genes in the TFCONES database.

**Figure 5:**
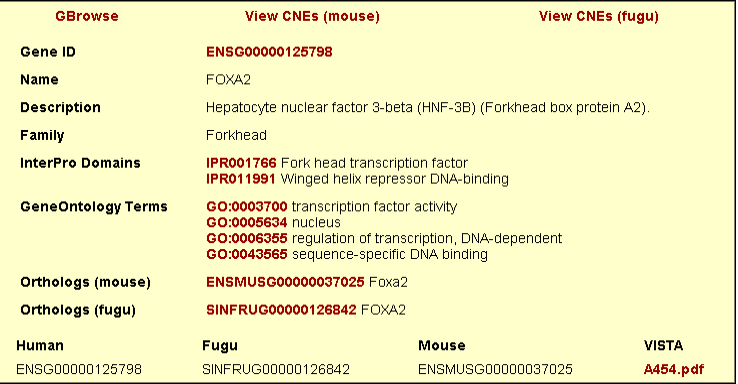
Gene record for forkhead transcription factor gene *FOXA2*.

**Figure 6:**
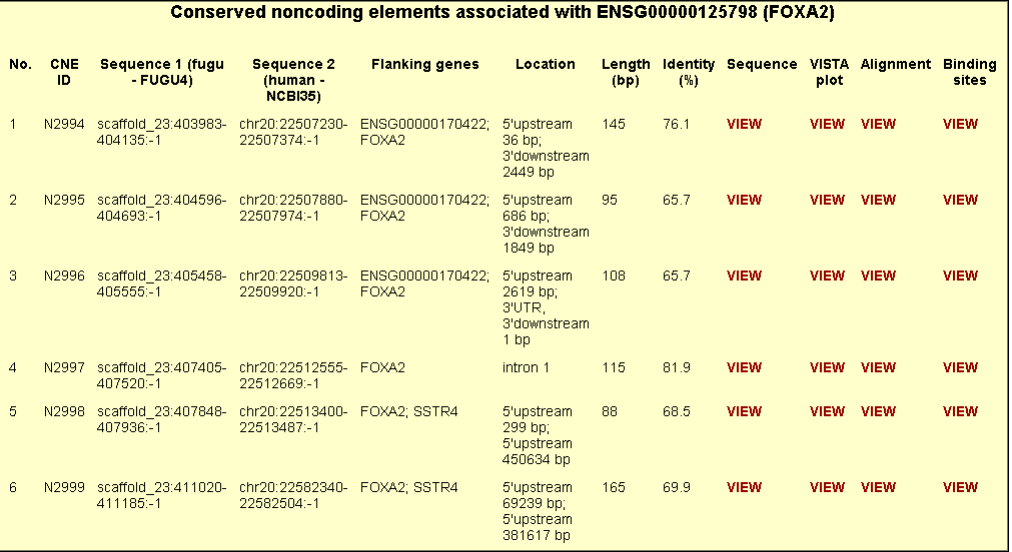
List of human-fugu CNEs associated with *FOXA2*.

**Figure 7:**
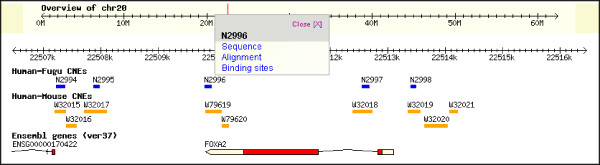
An image of the location of CNEs relative to the associated TF-encoding gene *FOXA2*.

We have created a database called TFCONES (Transcription Factor Genes & Associated COnserved Noncoding ElementS) that contains information about the list of TF-encoding genes in human, mouse and fugu genomes, their orthologous relationships, and details of the CNEs associated with them (Figs 4, 5 and 6). This set of human-mouse-fugu orthologous genes and their associated CNEs should be useful to researchers investigating the regulation and function of genes in mammals as well as in fishes. For every CNE, the data presented include the VISTA alignment, length, percentage identity, genomic location and the genes flanking the CNE. The users may search for specific human, mouse or fugu TF-encoding genes and retrieve the sequences of the human-mouse and human-fugu CNEs. The CNEs can be visualized in relation to the nearest TF-encoding gene using the GBrowse feature (Fig 7; [35]). The CNE database can also be BLAST-searched. To assist those interested in testing the function of potential TF binding sites within the human-fugu CNEs, we have predicted TF binding sites in the human-fugu CNEs using TESS [34]. While the human-fugu CNEs would be useful for scientists investigating evolutionarily conserved *cis*-regulatory elements that are likely to be shared by all vertebrates, the human-mouse CNEs should be of interest to researchers investigating *cis*-regulatory elements specific to mammals. In addition, TF-encoding genes that differentially express in certain tissues may be studied in greater detail to explore the gene regulatory networks active in those tissues. For instance, the CNEs that are associated with TF-encoding genes expressed in the central nervous system may be crucial in mediating expression precisely to distinct populations of neuronal and glial cells. These CNEs would be useful tools for marking cell-types through driving expression of reporter genes and will also act as useful reagents for targeting expression of therapeutic proteins to specific cell-types.

## Conclusion

TFs are the most crucial nodes in the gene regulatory networks that underlie the developmental plan and physiological systems of vertebrates. The evolutionarily conserved noncoding elements associated with TF-encoding genes in the TFCONES database represent a highly prioritized set of *cis*-regulatory sequences. These elements and the transcription factor binding sites identified in them should be useful for understanding the regulation of spatially restricted expression patterns of TF-encoding genes.

## Availability and Requirements

The TFCONES (Transcription Factor Genes & Associated COnserved Noncoding ElementS) database is freely accessible to academic and non-academic users at .

## List of abbreviations

CNE, conserved noncoding element; GO, Gene Ontology; TF, transcription factor

## Authors' contributions

BV and SB conceived and designed the project. APL and YY conducted the data analysis. APL and BV interpreted the data. APL built the TFCONES database. APL and BV wrote the manuscript. All authors read and approved the final manuscript.

## Supplementary Material

Additional data file 1 Human TF-encoding genes with more than one ortholog in fugu genome. Click here for file

Additional data file 2 Clusters of TF-encoding genes in the human genome. Click here for file

Additional data file 3 Clusters of TF-encoding genes in the mouse genome. Click here for file

Additional data file 4 Clusters of TF-encoding genes in the fugu genome. Click here for file

Additional data file 5 Conserved clusters of human, mouse and fugu TF-encoding genes. An asterisk indicates a human TF-encoding gene that has no ortholog in fugu but is located in a *Hox *conserved syntenic block. Click here for file

Additional data file 6 Distribution of lengths of (A) human-mouse and (B) human-fugu CNEs. Click here for file

Additional data file 7 Top twenty TF-encoding genes associated with the highest density of human-fugu CNEs. For genes that are part of conserved clusters, we averaged out the number or length of CNEs present in the whole cluster over the number of genes in that cluster. CNE density is defined as the number of bases located in CNEs per 100 bp of non-repetitive noncoding sequence in the longest orthologous fugu gene locus. Click here for file

Additional data file 8 Significantly over-represented and under-represented Gene Ontology terms (*P *< 0.01) of CNE-associated human TF-encoding genes. Group A denotes 385 of 389 CNE-associated TF-encoding genes with Gene Ontology annotation, while Group B denotes 804 of 816 orthologous TF-encoding genes with Gene Ontology annotation. *P*-values marked with a negative sign denote significant depletion. The analysis was carried out using GOstat (Beissbarth and Speed 2004). Click here for file
