# Supplementary material for: TFCONES: A database of vertebrate transcription factor-encoding genes and their associated conserved noncoding elements
Source: BMC Genomics. 2007 Nov 29;8:441. doi: 10.1186/1471-2164-8-441 (PMC2148067; doi:10.1186/1471-2164-8-441)
Supplement: Additional data file 1 — Human TF-encoding genes with more than one ortholog in fugu genome. [file 1471-2164-8-441-S1.doc]

Additional data file 1. Human TF-encoding genes with more than one ortholog in fugu genome.

| **Gene ID** | **Gene name** | **Description** |
| --- | --- | --- |
| ENSG00000172379 | *ARNT2* | Aryl hydrocarbon receptor nuclear translocator 2 |
| ENSG00000180828 | *BHLHB5* | basic helix-loop-helix domain containing, class B, 5 |
| ENSG00000105516 | *DBP* | D-site-binding protein |
| ENSG00000114861 | *FOXP1* | Forkhead box protein P1. |
| ENSG00000179348 | *GATA2* | Endothelial transcription factor GATA-2 |
| ENSG00000153807 | *HOXA10* | Homeobox protein Hox-A10 |
| ENSG00000005073 | *HOXA11* | Homeobox protein Hox-A11 |
| ENSG00000106031 | *HOXA13* | Homeobox protein Hox-A13 |
| ENSG00000105996 | *HOXA2* | Homeobox protein Hox-A2. |
| ENSG00000078399 | *HOXA9* | Homeobox protein Hox-A9 |
| ENSG00000120094 | *HOXB1* | Homeobox protein Hox-B1 |
| ENSG00000120093 | *HOXB3* | Homeobox protein Hox-B3 |
| ENSG00000120075 | *HOXB5* | Homeobox protein Hox-B5 |
| ENSG00000108511 | *HOXB6* | Homeobox protein Hox-B6 |
| ENSG00000128713 | *HOXD11* | Homeobox protein Hox-D11 |
| ENSG00000170166 | *HOXD4* | Homeobox protein Hox-D4 |
| ENSG00000128709 | *HOXD9* | Homeobox protein Hox-D9 |
| ENSG00000128604 | *IRF5* | Interferon regulatory factor 5 |
| ENSG00000092098 | *ISGF3G* | Transcriptional regulator ISGF3 gamma subunit |
| ENSG00000138136 | *LBX1* | Transcription factor LBX1. |
| ENSG00000103495 | *MAZ* | Myc-associated zinc finger protein |
| ENSG00000134138 | *MEIS2* | Homeobox protein Meis2 |
| ENSG00000188816 | *NP_005510.1* | Homeobox (H6 family) 2 |
| ENSG00000132326 | *PER2* | Period circadian protein 2. |
| ENSG00000137338 | *PGBD1* | piggyBac transposable element derived 1 |
| ENSG00000124216 | *SNAI1* | Zinc finger protein SNAI1 |
| ENSG00000100146 | *SOX10* | Transcription factor SOX-10. |
| ENSG00000168875 | *SOX14* | Transcription factor SOX-14. |
| ENSG00000182968 | *SOX1* | SOX-1 protein. |
| ENSG00000134595 | *SOX3* | Transcription factor SOX-3. |
| ENSG00000110693 | *SOX6* | Transcription factor SOX-6. |
| ENSG00000005513 | *SOX8* | Transcription factor SOX-8. |
| ENSG00000125398 | *SOX9* | Transcription factor SOX-9. |
| ENSG00000120948 | *TARDBP* | TAR DNA-binding protein 43 |
| ENSG00000105967 | *TFEC* | transcription factor EC isoform a |
| ENSG00000164853 | *XP_496843.1* | PREDICTED: similar to Uncx4.1 |
