# Supplementary material for: TFCONES: A database of vertebrate transcription factor-encoding genes and their associated conserved noncoding elements
Source: BMC Genomics. 2007 Nov 29;8:441. doi: 10.1186/1471-2164-8-441 (PMC2148067; doi:10.1186/1471-2164-8-441)
Supplement: Additional data file 2 — Clusters of TF-encoding genes in the human genome. [file 1471-2164-8-441-S2.doc]

Additional data file 2. Clusters of TF-encoding genes in the human genome.

| **No.** | **Gene IDs** | **Gene names (where available)** |
| --- | --- | --- |
| 12 genes |  |  |
| 1 | ENSG00000105991, ENSG00000105996, ENSG00000105997, ENSG00000197576, ENSG00000106004, ENSG00000106006, ENSG00000122592, ENSG00000078399, ENSG00000153807, ENSG00000005073, ENSG00000106031, ENSG00000106038 | *HOXA1, HOXA2, HOXA3, HOXA4, HOXA5, HOXA6, HOXA7, HOXA9, HOXA10, HOXA11, HOXA13, EVX1* |
|  |  |  |
| 10 genes |  |  |
| 1 | ENSG00000120094, ENSG00000173917, ENSG00000120093, ENSG00000182742, ENSG00000120075, ENSG00000108511, ENSG00000120087, ENSG00000120068, ENSG00000170689, ENSG00000159184 | *HOXB1, HOXB2, HOXB3, HOXB4, HOXB5, HOXB6, HOXB7, HOXB8, HOXB9, HOXB13* |
| 2 | ENSG00000174279, ENSG00000128714, ENSG00000170178, ENSG00000128713, ENSG00000128710, ENSG00000128709, ENSG00000175879, ENSG00000170166, ENSG00000128652, ENSG00000128645 | *EVX2, HOXD13, HOXD12, HOXD11, HOXD10, HOXD9, HOXD8, HOXD4, HOXD3, HOXD1* |
|  |  |  |
| 9 genes |  |  |
| 1 | ENSG00000123364, ENSG00000123407, ENSG00000123388, ENSG00000180818, ENSG00000180806, ENSG00000037965, ENSG00000197757, ENSG00000172789, ENSG00000198353 | *HOXC13, HOXC12, HOXC11, HOXC10, HOXC9, HOXC8, HOXC6, HOXC5, HOXC4* |
|  |  |  |
| 7 genes |  |  |
| 1 | ENSG00000167637, ENSG00000198453, ENSG00000197050, ENSG00000196967, ENSG00000197800, ENSG00000188283, ENSG00000181666 | *ZNF345, ZNF568, ZNF420, NP_954577.1, ZNF585B, ZNF383, HKR1* |
| 2 | ENSG00000161551, ENSG00000198093, ENSG00000176024, ENSG00000171032, ENSG00000197619, ENSG00000142556, ENSG00000196442 | *ZNF577, NP_075562.2, ZNF613, ZNF350, ZNF615, ZNF614, ZNF432* |
| 3 | ENSG00000152467, ENSG00000176293, ENSG00000121413, ENSG00000181894, ENSG00000171606, ENSG00000198131, ENSG00000083842 | *ZSCAN1, ZNF78L1, ZNF447, ZNF329, ZNF274, ZNF544, ZNF8* |
|  |  |  |
| 6 genes |  |  |
| 1 | ENSG00000124459, ENSG00000018607, ENSG00000159882, ENSG00000159885, ENSG00000178386, ENSG00000186019 | *ZNF45, ZNF221, ZNF155, ZNF222, ZNF223, ZNF27* |
|  |  |  |
| 5 genes |  |  |
| 1 | ENSG00000186812, ENSG00000186814, ENSG00000118267, ENSG00000172466, ENSG00000186496 | *ZNF397, Q86W11_HUMAN, ZNFEB_HUMAN, ZNF24, ZNF396* |
| 2 | ENSG00000172006, ENSG00000186300, ENSG00000172000, ENSG00000171970, ENSG00000175691 | *ZNF554, ZNF555, ZNF556, NP_775751.1, ZNF77* |
| 3 | ENSG00000196453, ENSG00000181220, ENSG00000133624, ENSG00000133619, ENSG00000181444 | *NP_056509.1, NP_689770.3, NP_079186.1, NP_115923.1, ZNF467* |
|  |  |  |
| 4 genes |  |  |
| 1 | ENSG00000196458, ENSG00000198393, ENSG00000198040, ENSG00000196387 | *ZNF605, ZNF26, ZNF84, ZNF140* |
| 2 | ENSG00000169951, ENSG00000197162, ENSG00000052835, ENSG00000156853 | *NP_219363.1, ZNF688, NP_689671.2, ZNF689* |
| 3 | ENSG00000188321, ENSG00000188629, ENSG00000174652, ENSG00000198028 | *ZNF559, ZNF177, ZNF266, ZNF560* |
| 4 | ENSG00000197372, ENSG00000196172, ENSG00000180081, ENSG00000183850 | *ZNF675, ZNF681, Q86Y87_HUMAN, ZNF539* |
| 5 | ENSG00000167562, ENSG00000123870, ENSG00000167766, ENSG00000175885 | *ZNF701, ZNF137, ZNF83, ZNF611* |
| 6 | ENSG00000170949, ENSG00000170954, ENSG00000197937, ENSG00000197497 | *KR18_HUMAN, ZNF415, ZNF347, NP_079009.2* |
| 7 | ENSG00000131845, ENSG00000152433, ENSG00000188785, ENSG00000186272 | *ZNF304, ZNF547, ZNF548, NP_008890.1* |
| 8 | ENSG00000183647, ENSG00000166680, ENSG00000121417, ENSG00000180532 | *ZNF530, ZNF134, ZNF211, ZSCAN4* |
| 9 | ENSG00000171574, ENSG00000131849, ENSG00000083812, ENSG00000083838 | *ZNF584, ZNF132, ZNF324, ZNF446* |
| 10 | ENSG00000197657, ENSG00000189298, ENSG00000158691, ENSG00000187987 | *ZNF323, ZNF306, ZNF96, Q96KW0_HUMAN* |
| 11 | ENSG00000197362, ENSG00000197024, ENSG00000170265, ENSG00000170260 | *NP_689624.1, ZNF398, ZNF282, ZNF212* |
|  |  |  |
| 3 genes |  |  |
| 1 | ENSG00000197472, ENSG00000135747, ENSG00000188295 | *ZNF695, ZNF670, ZNF669* |
| 2 | ENSG00000198105, ENSG00000175395, ENSG00000189180 | *ZNF248, ZNF25, ZNF33A* |
| 3 | ENSG00000196793, ENSG00000198298, ENSG00000169740 | *ZNF239, ZNF485, ZNF32* |
| 4 | ENSG00000184302, ENSG00000126778, ENSG00000100625 | *SIX6, SIX1, SIX4* |
| 5 | ENSG00000130182, ENSG00000122386, ENSG00000085644 | *ZNF206, ZNF205, ZNF213* |
| 6 | ENSG00000177508, ENSG00000176842, ENSG00000159387 | *IRX3, IRX5, IRX6* |
| 7 | ENSG00000173757, ENSG00000126561, ENSG00000168610 | *STAT5B, STAT5A, STAT3* |
| 8 | ENSG00000132010, ENSG00000196646, ENSG00000197857 | *ZNF625, ZNF136, ZNF44* |
| 9 | ENSG00000089335, ENSG00000153896, ENSG00000168661 | *ZNF302, ZNF599, ZNF30* |
| 10 | ENSG00000161298, ENSG00000197808, ENSG00000189042 | *ZNF382, GIOT1_HUMAN, ZNF567* |
| 11 | ENSG00000180479, ENSG00000171817, ENSG00000120784 | *ZNF571, ZNF540, ZFP30_HUMAN* |
| 12 | ENSG00000159904, ENSG00000159905, ENSG00000167380 | *ZNF225, ZNF234, ZNF226* |
| 13 | ENSG00000159915, ENSG00000159917, ENSG00000062370 | *ZNF233, ZNF235, ZNF228* |
| 14 | ENSG00000196214, ENSG00000198464, ENSG00000167554 | *NP_001010851.1, ZNF480, ZNF610* |
| 15 | ENSG00000105066, ENSG00000171443, ENSG00000179922 | *FIZ1_HUMAN, ZNF524, NP_976308.1* |
| 16 | ENSG00000197025, ENSG00000018869, ENSG00000198440 | *ZNF542, ZNF582, ZNF583* |
| 17 | ENSG00000121406, ENSG00000105132, ENSG00000083817 | *ZNF549, ZNF550, ZNF416* |
| 18 | ENSG00000125816, ENSG00000125820, ENSG00000125813 | *NKX2-4, NKX2-2, PAX1* |
| 19 | ENSG00000101115, ENSG00000020256, ENSG00000182463 | *SALL4, ZFP64, ZNF218* |
| 20 | ENSG00000185219, ENSG00000178917, ENSG00000196345 | *ZNF445, Q6ZMS4_HUMAN, ZNF167* |
| 21 | ENSG00000186448, ENSG00000169981, ENSG00000196653 | *ZNF197, ZNF35, ZNF502* |
| 22 | ENSG00000164379, ENSG00000137273, ENSG00000054598 | *FOXQ1, FOXF2, FOXC1* |
| 23 | ENSG00000197279, ENSG00000196812, ENSG00000198315 | *ZNF165, ZNF435, ZNF192* |
| 24 | ENSG00000106261, ENSG00000166529, ENSG00000166526 | *ZKSCAN1, ZNF38, ZNF3* |
| 25 | ENSG00000137090, ENSG00000064218, ENSG00000173253 | *DMRT1, DMRT3, DMRT2* |
| 26 | ENSG00000157965, ENSG00000187754, ENSG00000157950 | *SSX8, SSX2, SSX2* |
|  |  |  |
| 2 genes |  |  |
| 1 | ENSG00000007968, ENSG00000117318 | *E2F2, ID3* |
| 2 | ENSG00000176083, ENSG00000131914 | *ZNF683, LIN28* |
| 3 | ENSG00000186790, ENSG00000186564 | *FOXE3, FOXD2* |
| 4 | ENSG00000143006, ENSG00000174332 | *DMRTB1, GLIS1* |
| 5 | ENSG00000172018, ENSG00000117036 | *NP_001004341.1, ETV3* |
| 6 | ENSG00000162761, ENSG00000143171 | *LMX1A, RXRG* |
| 7 | ENSG00000165512, ENSG00000183297 | *ZNF22, NM_001012729.1* |
| 8 | ENSG00000107807, ENSG00000138136 | *TLX1, LBX1* |
| 9 | ENSG00000188620, ENSG00000188816 | *XP_291716.5, NP_005510.1* |
| 10 | ENSG00000171794, ENSG00000151650 | *UTF1, VENTX* |
| 11 | ENSG00000149054, ENSG00000149050 | *ZNF215, ZNF214* |
| 12 | ENSG00000135374, ENSG00000135373 | *ELF5, EHF* |
| 13 | ENSG00000134954, ENSG00000151702 | *ETS1, FLI1* |
| 14 | ENSG00000166080, ENSG00000043039 | *ZNF123, BARX2* |
| 15 | ENSG00000170322, ENSG00000170325 | *NFRKB, PRDM10* |
| 16 | ENSG00000078246, ENSG00000197905 | *TULP3, TEAD4* |
| 17 | ENSG00000135457, ENSG00000184271 | *TFCP2, POU6F1* |
| 18 | ENSG00000170374, ENSG00000185591 | *SP7, SP1* |
| 19 | ENSG00000111046, ENSG00000111049 | *MYF6, MYF5* |
| 20 | ENSG00000169840, ENSG00000139515 | *GSH1, IPF1* |
| 21 | ENSG00000139800, ENSG00000043355 | *ZIC5, ZIC2* |
| 22 | ENSG00000100644, ENSG00000023608 | *HIF1A, SNAPC1* |
| 23 | ENSG00000140044, ENSG00000156127 | *NP_569736.1, BATF* |
| 24 | ENSG00000198146, ENSG00000179437 | *NP_054825.2, NANOGP8* |
| 25 | ENSG00000137834, ENSG00000166949 | *SMAD6, SMAD3* |
| 26 | ENSG00000103343, ENSG00000167981 | *ZNF174, ZNF597* |
| 27 | ENSG00000090447, ENSG00000126603 | *TFAP4, GLIS2* |
| 28 | ENSG00000180035, ENSG00000179965 | *ZNF553, NP_057727.1* |
| 29 | ENSG00000167394, ENSG00000167395 | *ZNF668, ZNF646* |
| 30 | ENSG00000167377, ENSG00000157429 | *ZNF23, ZNF19* |
| 31 | ENSG00000176692, ENSG00000176678 | *FOXC2, FOXL1* |
| 32 | ENSG00000180787, ENSG00000167840 | *ZFP3, ZNF232* |
| 33 | ENSG00000179111, ENSG00000179094 | *HES7, PER1* |
| 34 | ENSG00000108452, ENSG00000187607 | *ZNF29, ZNF286* |
| 35 | ENSG00000132130, ENSG00000108270 | *LHX1, AATF* |
| 36 | ENSG00000126351, ENSG00000126368 | *THRA, NR1D1* |
| 37 | ENSG00000175832, ENSG00000005102 | *ETV4, MEOX1* |
| 38 | ENSG00000108813, ENSG00000064195 | *DLX4, DLX3* |
| 39 | ENSG00000171469, ENSG00000171466 | *ZNF561, ZNF562* |
| 40 | ENSG00000197332, ENSG00000197933 | *NP_001013713.1, ZF36_HUMAN* |
| 41 | ENSG00000177599, ENSG00000171295 | *ZNF491, ZNF440* |
| 42 | ENSG00000188033, ENSG00000173875 | *ZNF490, NP_699189.1* |
| 43 | ENSG00000008441, ENSG00000104903 | *NFIX, LYL1* |
| 44 | ENSG00000181896, ENSG00000105708 | *ZNF101, ZNF14* |
| 45 | ENSG00000081665, ENSG00000197124 | *ZNF93, ZNF682* |
| 46 | ENSG00000105750, ENSG00000118620 | *ZNF85, ZNF430* |
| 47 | ENSG00000197020, ENSG00000198521 | *ZNF100, ZNF43* |
| 48 | ENSG00000105699, ENSG00000105698 | *LSR, USF2* |
| 49 | ENSG00000181007, ENSG00000186017 | *ZNF545, ZNF566* |
| 50 | ENSG00000105732, ENSG00000028277 | *ZNF574, POU2F2* |
| 51 | ENSG00000105722, ENSG00000079432 | *ERF, CIC* |
| 52 | ENSG00000178928, ENSG00000105392 | *TPRX1, CRX* |
| 53 | ENSG00000198304, ENSG00000167555 | *ZNF528, Q96LN7_HUMAN* |
| 54 | ENSG00000198346, ENSG00000130844 | *NP_612381.1, ZNF331* |
| 55 | ENSG00000179909, ENSG00000083814 | *ZNF154, ZNF671* |
| 56 | ENSG00000138083, ENSG00000170577 | *SIX3, SIX2* |
| 57 | ENSG00000144026, ENSG00000163067 | *ZNF514, ZNF2* |
| 58 | ENSG00000074047, ENSG00000115112 | *GLI2, TFCP2L1* |
| 59 | ENSG00000144355, ENSG00000115844 | *DLX1, DLX2* |
| 60 | ENSG00000115415, ENSG00000138378 | *STAT1, STAT4* |
| 61 | ENSG00000118263, ENSG00000118260 | *KLF7, CREB1* |
| 62 | ENSG00000144485, ENSG00000132326 | *HES6, PER2* |
| 63 | ENSG00000125878, ENSG00000172070 | *TCF15, SRXN1* |
| 64 | ENSG00000124226, ENSG00000124216 | *ZNF313, SNAI1* |
| 65 | ENSG00000125520, ENSG00000130584 | *SLC2A4RG, BTBD4* |
| 66 | ENSG00000185569, ENSG00000184221 | *OLIG2, OLIG1* |
| 67 | ENSG00000157554, ENSG00000157557 | *ERG, ETS2* |
| 68 | ENSG00000151090, ENSG00000077092 | *THRB, RARB* |
| 69 | ENSG00000187098, ENSG00000114861 | *MITF, FOXP1* |
| 70 | ENSG00000174963, ENSG00000152977 | *ZIC4, ZIC1* |
| 71 | ENSG00000131127, ENSG00000186777 | *ZNF141, ABCA11* |
| 72 | ENSG00000113430, ENSG00000170561 | *IRX4, IRX2* |
| 73 | ENSG00000198939, ENSG00000178187 | *ZFP2, ZNF454* |
| 74 | ENSG00000137185, ENSG00000187626 | *ZNF193, ZNF307* |
| 75 | ENSG00000197062, ENSG00000137338 | *ZNF187, PGBD1* |
| 76 | ENSG00000137310, ENSG00000137369 | *TCF19, POU5F1* |
| 77 | ENSG00000008197, ENSG00000008196 | *TFAP2D, TFAP2B* |
| 78 | ENSG00000196247, ENSG00000197008 | *ZNF588, ZNF138* |
| 79 | ENSG00000198039, ENSG00000152926 | *ZNF273, NP_056936.1* |
| 80 | ENSG00000006377, ENSG00000105880 | *DLX6, DLX5* |
| 81 | ENSG00000160908, ENSG00000196652 | *ZNF394, ZFP95* |
| 82 | ENSG00000197343, ENSG00000197037 | *ZNF655, ZNF498* |
| 83 | ENSG00000167034, ENSG00000180053 | *NKX3-1, XP_070619.8* |
| 84 | ENSG00000104313, ENSG00000178860 | *EYA1, MSC* |
| 85 | ENSG00000164749, ENSG00000091656 | *HNF4G, ZFHX4* |
| 86 | ENSG00000120963, ENSG00000083307 | *ZNF706, GRHL2* |
| 87 | ENSG00000181638, ENSG00000185730 | *GLI4, ZNF696* |
| 88 | ENSG00000196150, ENSG00000170631 | *ZNF250, ZNF16* |
| 89 | ENSG00000197550, ENSG00000184659 | *ZNF72, FOXD4L4* |
| 90 | ENSG00000081386, ENSG00000196597 | *ZNF510* |
| 91 | ENSG00000136931, ENSG00000148200 | *NR5A1, NR6A1* |
| 92 | ENSG00000107164, ENSG00000130711 | *FUBP3, PRDM12* |
| 93 | ENSG00000147117, ENSG00000147124 | *ZNF157, ZNF41* |
| 94 | ENSG00000147118, ENSG00000171483 | *ZNF630, SSX6* |
| 95 | ENSG00000165584, ENSG00000198946 | *SSX3, SSX4* |
| 96 | ENSG00000184911, ENSG00000159123 | *DMRTC1, DMRTC1* |
| 97 | ENSG00000101883, ENSG00000131721 | *PEPP1_HUMAN, PEPP2_HUMAN* |
| 98 | ENSG00000186376, ENSG00000173275 | *ZNF75, ZNF449* |
| 99 | ENSG00000188841, ENSG00000022267 | *XP_066752.1, FHL1* |
| 100 | ENSG00000063587, ENSG00000189420 | *ZNF275, XP_066859.3* |
| 101 | ENSG00000067646, ENSG00000176679 | *ZFY, TGIF2LY* |
