# Supplementary material for: TFCONES: A database of vertebrate transcription factor-encoding genes and their associated conserved noncoding elements
Source: BMC Genomics. 2007 Nov 29;8:441. doi: 10.1186/1471-2164-8-441 (PMC2148067; doi:10.1186/1471-2164-8-441)
Supplement: Additional data file 3 — Clusters of TF-encoding genes in the mouse genome. [file 1471-2164-8-441-S3.doc]

Additional data file 3. Clusters of TF-encoding genes in the mouse genome.

| **No.** | **Gene IDs** | **Gene names (where available)** |
| --- | --- | --- |
| 12 genes |  |  |
| 1 | ENSMUSG00000029844, ENSMUSG00000014704, ENSMUSG00000059723, ENSMUSG00000000942, ENSMUSG00000038253, ENSMUSG00000043219, ENSMUSG00000038236, ENSMUSG00000038227, ENSMUSG00000000938, ENSMUSG00000038210, ENSMUSG00000038203, ENSMUSG00000005503 | *Hoxa1, Hoxa2, 2700086A05Rik, Hoxa4, Hoxa5, Hoxa6, Hoxa7, Hoxa9, Hoxa10, Hoxa11, Hoxa13, Evx1* |
|  |  |  |
| 10 genes |  |  |
| 1 | ENSMUSG00000049604, ENSMUSG00000020875, ENSMUSG00000056648, ENSMUSG00000038721, ENSMUSG00000000690, ENSMUSG00000038700, ENSMUSG00000038692, ENSMUSG00000048763, ENSMUSG00000047830, ENSMUSG00000018973 | *Hoxb13, Hoxb9, Hoxb8, Hoxb7, Hoxb6, Hoxb5, Hoxb4, Hoxb3, Hoxb2, Hoxb1* |
| 2 | ENSMUSG00000001815, ENSMUSG00000001819, ENSMUSG00000001823, ENSMUSG00000042499, ENSMUSG00000050368, ENSMUSG00000043342, ENSMUSG00000027102, ENSMUSG00000042464, ENSMUSG00000042464, ENSMUSG00000042448 | *Evx2, Hoxd13, Hoxd12, Hoxd11, Hoxd10, Hoxd9, Hoxd8, Hoxd4, Hoxd3, Hoxd1* |
|  |  |  |
| 9 genes |  |  |
| 1 | ENSMUSG00000001655, ENSMUSG00000050328, ENSMUSG00000001656, ENSMUSG00000022484, ENSMUSG00000036139, ENSMUSG00000001657, ENSMUSG00000001661, ENSMUSG00000022485, ENSMUSG00000022486 | *Hoxc13, Hoxc12, HXC12_MOUSE, Hoxc10, Hoxc9, Hoxc8, Hoxc6, Hoxc5, Hoxc4* |
|  |  |  |
| 8 genes |  |  |
| 1 | ENSMUSG00000051499, ENSMUSG00000062519, ENSMUSG00000025821, ENSMUSG00000052763, ENSMUSG00000045466, ENSMUSG00000057691, ENSMUSG00000042810, ENSMUSG00000068551 | *A730012O14Rik, Zfp398, Zfp282, Zfp212, AI894139, Q3U133_MOUSE, A930040G15Rik, Zfp467* |
|  |  |  |
| 5 genes |  |  |
| 1 | ENSMUSG00000034001, ENSMUSG00000057894, ENSMUSG00000058638, ENSMUSG00000060397, ENSMUSG00000054715 | *Q52KH8_MOUSE, Zfp329, Zfp110, Zfp128, Hkr2* |
|  |  |  |
| 4 genes |  |  |
| 1 | ENSMUSG00000036721, ENSMUSG00000021327, ENSMUSG00000055313, ENSMUSG00000022228 | *Zfp96, Zfp306, 4921509E05Rik, Zfp187* |
| 2 | ENSMUSG00000062862, ENSMUSG00000048928, ENSMUSG00000030486, ENSMUSG00000055305 | *Zfp111, Zfp109, Zfp108, Zfp93* |
|  |  |  |
| 3 genes |  |  |
| 1 | ENSMUSG00000026380, ENSMUSG00000051835, ENSMUSG00000048402 | *Tcfcp2l1, Gli2, XP_136212.4* |
| 2 | ENSMUSG00000015843, ENSMUSG00000026686, ENSMUSG00000052534 | *Rxrg, Lmx1a, Pbx1* |
| 3 | ENSMUSG00000020919, ENSMUSG00000004043, ENSMUSG00000004040 | *Stat5b, Stat5a, Stat3* |
| 4 | ENSMUSG00000001496, ENSMUSG00000058669, ENSMUSG00000001497 | *Titf1, Nkx2-9, Pax9* |
| 5 | ENSMUSG00000021099, ENSMUSG00000051367, ENSMUSG00000034460 | *Six6, Six1, Six4* |
| 6 | ENSMUSG00000021250, ENSMUSG00000034271, ENSMUSG00000034266 | *Fos, Jundm2, Batf* |
| 7 | ENSMUSG00000024837, ENSMUSG00000042372, ENSMUSG00000048138 | *Dmrt1, Dmrt3, Dmrt2* |
| 8 | ENSMUSG00000041459, ENSMUSG00000028977, ENSMUSG00000043632 | *Tardbp, Casz1, BC035954* |
| 9 | ENSMUSG00000029729, ENSMUSG00000037017, ENSMUSG00000037007 | *Zkscan1, Zipro1, Zfp113* |
| 10 | ENSMUSG00000029627, ENSMUSG00000055991, ENSMUSG00000007812 | *Zfp99, Zfp95, Zfp655* |
| 11 | ENSMUSG00000053129, ENSMUSG00000029644, ENSMUSG00000029646 | *Gsh1, Ipf1, Cdx2* |
| 12 | ENSMUSG00000057101, ENSMUSG00000052675, ENSMUSG00000047603 | *Zfp180, Zfp112, Zfp235* |
| 13 | ENSMUSG00000058402, ENSMUSG00000062040, ENSMUSG00000059975 | *B230312I18Rik, Zfp27, Zfp74* |
| 14 | ENSMUSG00000058447, ENSMUSG00000049421, ENSMUSG00000063979 | *Zfp82, Zfp260, Q3UYY9_MOUSE* |
| 15 | ENSMUSG00000056592, ENSMUSG00000030469, ENSMUSG00000055102 | *BC043301, C630016O21Rik, 4933405K07Rik* |
| 16 | ENSMUSG00000045757, ENSMUSG00000045251, ENSMUSG00000048921 | *8030466O12Rik, Zfp688, Zfp689* |
| 17 | ENSMUSG00000047036, ENSMUSG00000063488, ENSMUSG00000057895 | *Zfp445, Zfp105* |
|  |  |  |
| 2 genes |  |  |
| 1 | ENSMUSG00000025932, ENSMUSG00000025930 | *Eya1, Msc* |
| 2 | ENSMUSG00000042596, ENSMUSG00000025927 | *Tcfap2d, Tcfap2b* |
| 3 | ENSMUSG00000045336, ENSMUSG00000038331 | *Hsfy2, Satb2* |
| 4 | ENSMUSG00000067071, ENSMUSG00000055866 | *Hes6, Per2* |
| 5 | ENSMUSG00000000435, ENSMUSG00000035923 | *Myf5, Myf6* |
| 6 | ENSMUSG00000048728, ENSMUSG00000049321 | *Zfp454, Zfp2* |
| 7 | ENSMUSG00000064145, ENSMUSG00000020472 | *XP_484030.1, Zfp496* |
| 8 | ENSMUSG00000005267, ENSMUSG00000047342 | *Zfp287, Zfp286* |
| 9 | ENSMUSG00000020893, ENSMUSG00000023781 | *Per1, Hes7* |
| 10 | ENSMUSG00000001510, ENSMUSG00000020871 | *Dlx3, Dlx4* |
| 11 | ENSMUSG00000058756, ENSMUSG00000020889 | *Thra, Nr1d1* |
| 12 | ENSMUSG00000017724, ENSMUSG00000001493 | *Etv4, Meox1* |
| 13 | ENSMUSG00000020644, ENSMUSG00000020642 | *Id2, Rnf144* |
| 14 | ENSMUSG00000038402, ENSMUSG00000050295 | *Foxf2, Foxc1* |
| 15 | ENSMUSG00000001504, ENSMUSG00000021604 | *Irx2, Irx4* |
| 16 | ENSMUSG00000062431, ENSMUSG00000034522 | *Zfp395* |
| 17 | ENSMUSG00000044186, ENSMUSG00000022061 | *Nkx2-6, Nkx3-1* |
| 18 | ENSMUSG00000005148, ENSMUSG00000022060 | *Klf5, Klf12* |
| 19 | ENSMUSG00000041703, ENSMUSG00000061524 | *Zic5* |
| 20 | ENSMUSG00000009733, ENSMUSG00000009739 | *Tcfcp2, Pou6f1* |
| 21 | ENSMUSG00000060284, ENSMUSG00000001280 | *Sp7, Sp1* |
| 22 | ENSMUSG00000054939, ENSMUSG00000039789 | *XP_358903.3* |
| 23 | ENSMUSG00000005718, ENSMUSG00000014303 | *Tcfap4, Glis2* |
| 24 | ENSMUSG00000040732, ENSMUSG00000022895 | *Erg, Ets2* |
| 25 | ENSMUSG00000024406, ENSMUSG00000050410 | *NP_038661.1, Tcf19* |
| 26 | ENSMUSG00000038805, ENSMUSG00000024134 | *Six3, Six2* |
| 27 | ENSMUSG00000024276, ENSMUSG00000024274 | *Zfp397, XP_283527.1* |
| 28 | ENSMUSG00000025215, ENSMUSG00000025216 | *Tlx1, Lbx1* |
| 29 | ENSMUSG00000023094, ENSMUSG00000026735 | *Msrb2, Ptf1a* |
| 30 | ENSMUSG00000026751, ENSMUSG00000063972 | *Nr5a1, Nr6a1* |
| 31 | ENSMUSG00000041911, ENSMUSG00000023391 | *Dlx1, Dlx2* |
| 32 | ENSMUSG00000012350, ENSMUSG00000027186 | *Ehf, Elf5* |
| 33 | ENSMUSG00000059962, ENSMUSG00000050619 | *XP_619803.1, Zfp690* |
| 34 | ENSMUSG00000054160, ENSMUSG00000027434 | *Nkx2-4, Nkx2-2* |
| 35 | ENSMUSG00000006418, ENSMUSG00000042821 | *Zfp313, Snai1* |
| 36 | ENSMUSG00000027547, ENSMUSG00000027551 | *Sall4, NP_033590.1* |
| 37 | ENSMUSG00000003382, ENSMUSG00000042789 | *Etv3* |
| 38 | ENSMUSG00000034762, ENSMUSG00000028610 | *Glis1, Dmrtb1* |
| 39 | ENSMUSG00000055210, ENSMUSG00000044518 | *Foxd2, Foxe3* |
| 40 | ENSMUSG00000049878, ENSMUSG00000056322 | *Rlf* |
| 41 | ENSMUSG00000050966, ENSMUSG00000049410 | *Lin28* |
| 42 | ENSMUSG00000007872, ENSMUSG00000018983 | *Id3, E2f2* |
| 43 | ENSMUSG00000053344, ENSMUSG00000066036 | *1810009A16Rik, 1810009A16Rik* |
| 44 | ENSMUSG00000067222, ENSMUSG00000060721 | *Nfxl1, Nfxl1* |
| 45 | ENSMUSG00000018604, ENSMUSG00000018263 | *Tbx3, Tbx5* |
| 46 | ENSMUSG00000029754, ENSMUSG00000029755 | *Dlx6, Dlx5* |
| 47 | ENSMUSG00000038157, ENSMUSG00000053007 | *Creb5* |
| 48 | ENSMUSG00000035158, ENSMUSG00000030067 | *Mitf, Foxp1* |
| 49 | ENSMUSG00000059689, ENSMUSG00000042097 | *Zfp637, Zfp239* |
| 50 | ENSMUSG00000061374, ENSMUSG00000051184 | *Fiz1, Zfp524* |
| 51 | ENSMUSG00000043290, ENSMUSG00000055633 | *4632433K11Rik, Zfp580* |
| 52 | ENSMUSG00000004500, ENSMUSG00000033961 | *Zfp324, Zfp446* |
| 53 | ENSMUSG00000001988, ENSMUSG00000058230 | *Npas1, 6430596G11Rik* |
| 54 | ENSMUSG00000045252, ENSMUSG00000008496 | *Zfp574, Pou2f2* |
| 55 | ENSMUSG00000040857, ENSMUSG00000005442 | *Erf, Cic* |
| 56 | ENSMUSG00000058239, ENSMUSG00000001247 | *Usf2, Lisch7* |
| 57 | ENSMUSG00000021217, ENSMUSG00000043456 | *Zfp537, Zfp536* |
| 58 | ENSMUSG00000030757, ENSMUSG00000066187 | *Zfp694, Zfp694* |
| 59 | ENSMUSG00000045598, ENSMUSG00000054716 | *Zfp553, G630024C07Rik* |
| 60 | ENSMUSG00000040148, ENSMUSG00000050100 | *Hmx3, Hmx2* |
| 61 | ENSMUSG00000034041, ENSMUSG00000001911 | *Lyl1, Nfix* |
| 62 | ENSMUSG00000031737, ENSMUSG00000031738 | *Irx5, Irx6* |
| 63 | ENSMUSG00000041515, ENSMUSG00000042812 | *Irf8, Foxf1a* |
| 64 | ENSMUSG00000063108, ENSMUSG00000059475 | *Zfp26, Zfp426* |
| 65 | ENSMUSG00000042496, ENSMUSG00000042185 | *Prdm10, Nfrkb* |
| 66 | ENSMUSG00000016087, ENSMUSG00000032035 | *Fli1, ETS1_MOUSE* |
| 67 | ENSMUSG00000032402, ENSMUSG00000036867 | *Smad3, Smad6* |
| 68 | ENSMUSG00000032368, ENSMUSG00000036972 | *Zic1, Zic4* |
| 69 | ENSMUSG00000031365, ENSMUSG00000031374 | *NP_113682.1, Zfp92* |
| 70 | ENSMUSG00000025529, ENSMUSG00000052063 | *A230078I01Rik* |
