# Supplementary material for: TFCONES: A database of vertebrate transcription factor-encoding genes and their associated conserved noncoding elements
Source: BMC Genomics. 2007 Nov 29;8:441. doi: 10.1186/1471-2164-8-441 (PMC2148067; doi:10.1186/1471-2164-8-441)
Supplement: Additional data file 4 — Clusters of TF-encoding genes in the fugu genome. [file 1471-2164-8-441-S4.doc]

Additional data file 4. Clusters of TF-encoding genes in the fugu genome.

| **No.** | **Gene IDs** | **Gene names (where available)** |
| --- | --- | --- |
| 10 genes |  |  |
| 1 | SINFRUG00000149627, SINFRUG00000157594, SINFRUG00000149628, SINFRUG00000149629, SINFRUG00000149630, SINFRUG00000149637, SINFRUG00000149640, SINFRUG00000157392, SINFRUG00000149643, SINFRUG00000165271 | *HoxA1a, HoxA2b, HoxA3a, HoxA4a, HoxA5a, HoxA9a, HoxA10a, HoxA11a, HoxA13a, Evx1* |
|  |  |  |
| 9 genes |  |  |
| 1 | SINFRUG00000163355, SINFRUG00000160069, SINFRUG00000136620, SINFRUG00000165134, SINFRUG00000136624, SINFRUG00000136631, SINFRUG00000157209, SINFRUG00000136637, SINFRUG00000136635 | *HoxB13a, HoxB9a, HoxB8a, HoxB6a, HoxB5a, HoxB4a, HoxB3a, HoxB2a, HoxB1a* |
| 2 | SINFRUG00000146324, SINFRUG00000146325, SINFRUG00000146326, SINFRUG00000146328, SINFRUG00000146331, SINFRUG00000146333, SINFRUG00000149367, SINFRUG00000164408, SINFRUG00000149366 | *HoxC4a, HoxC5a, HoxC6a, HoxC8a, HoxC9a, HoxC10a, HoxC11a, HoxC12a, HoxC13a* |
|  |  |  |
| 7 genes |  |  |
| 1 | SINFRUG00000124774, SINFRUG00000124775, SINFRUG00000124776, SINFRUG00000124778, SINFRUG00000162044, SINFRUG00000120943, SINFRUG00000158421 | *HoxD3a, HoxD4a, HoxD9a, HoxD10a, HoxD11a, HoxD12a, Evx2* |
|  |  |  |
| 5 genes |  |  |
| 1 | SINFRUG00000138063, SINFRUG00000138062, SINFRUG00000138061, SINFRUG00000138060, SINFRUG00000132089 | *HoxA2b, HoxA9b, HoxA10b, HoxA11b, HoxA13b* |
|  |  |  |
| 4 genes |  |  |
| 1 | SINFRUG00000159847, SINFRUG00000163469, SINFRUG00000132418, SINFRUG00000132419 | *EIF-4A, Q90513_FUGRU, FOS* |
| 2 | SINFRUG00000152798, SINFRUG00000162209, SINFRUG00000161182, SINFRUG00000124288 |  |
| 3 | SINFRUG00000156875, SINFRUG00000132774, SINFRUG00000161068, SINFRUG00000131120 | *HoxB6b, HoxB5b, HoxB3b, HoxB1b* |
| 4 | SINFRUG00000123771, SINFRUG00000123764, SINFRUG00000161782, SINFRUG00000123755 |  |
|  |  |  |
| 3 genes |  |  |
| 1 | SINFRUG00000129400, SINFRUG00000129398, SINFRUG00000129395 | *dmrt2, dmrt3, dmrt1* |
| 2 | SINFRUG00000148860, SINFRUG00000161134, SINFRUG00000145617 |  |
| 3 | SINFRUG00000145501, SINFRUG00000145503, SINFRUG00000125776 |  |
| 4 | SINFRUG00000124576, SINFRUG00000124575, SINFRUG00000124573 |  |
| 5 | SINFRUG00000137684, SINFRUG00000133296, SINFRUG00000133300 |  |
| 6 | SINFRUG00000161692, SINFRUG00000156364, SINFRUG00000162165 | *HoxD11b, HoxD9b, HoxD4b* |
| 7 | SINFRUG00000135681, SINFRUG00000135691, SINFRUG00000135695 |  |
| 8 | SINFRUG00000148198, SINFRUG00000148199, SINFRUG00000161740 |  |
| 9 | SINFRUG00000145191, SINFRUG00000123903, SINFRUG00000155313 |  |
| 10 | SINFRUG00000124258, SINFRUG00000124257, SINFRUG00000124252 |  |
| 11 | SINFRUG00000136495, SINFRUG00000160630, SINFRUG00000140913 |  |
| 12 | SINFRUG00000164529, SINFRUG00000144886, SINFRUG00000161675 |  |
|  |  |  |
| 2 genes |  |  |
| 1 | SINFRUG00000125144, SINFRUG00000125143 |  |
| 2 | SINFRUG00000126134, SINFRUG00000146762 |  |
| 3 | SINFRUG00000129634, SINFRUG00000129633 |  |
| 4 | SINFRUG00000137802, SINFRUG00000165186 |  |
| 5 | SINFRUG00000120430, SINFRUG00000120428 |  |
| 6 | SINFRUG00000130983, SINFRUG00000163564 |  |
| 7 | SINFRUG00000164263, SINFRUG00000141792 |  |
| 8 | SINFRUG00000149958, SINFRUG00000149959 |  |
| 9 | SINFRUG00000135010, SINFRUG00000163138 |  |
| 10 | SINFRUG00000123819, SINFRUG00000159174 | *myf5, mrf4* |
| 11 | SINFRUG00000138887, SINFRUG00000161016 |  |
| 12 | SINFRUG00000123879, SINFRUG00000138071 |  |
| 13 | SINFRUG00000147877, SINFRUG00000158918 |  |
| 14 | SINFRUG00000164825, SINFRUG00000140973 | *Sox4* |
| 15 | SINFRUG00000126933, SINFRUG00000126931 |  |
| 16 | SINFRUG00000156291, SINFRUG00000133369 | *ND2* |
| 17 | SINFRUG00000136433, SINFRUG00000162794 |  |
| 18 | SINFRUG00000160010, SINFRUG00000123351 |  |
| 19 | SINFRUG00000141521, SINFRUG00000127307 |  |
| 20 | SINFRUG00000127293, SINFRUG00000127292 |  |
| 21 | SINFRUG00000122591, SINFRUG00000122597 |  |
| 22 | SINFRUG00000126064, SINFRUG00000126067 |  |
| 23 | SINFRUG00000123039, SINFRUG00000161948 |  |
| 24 | SINFRUG00000157202, SINFRUG00000135018 |  |
| 25 | SINFRUG00000145593, SINFRUG00000141467 |  |
| 26 | SINFRUG00000156741, SINFRUG00000151780 |  |
| 27 | SINFRUG00000128384, SINFRUG00000148422 |  |
| 28 | SINFRUG00000162349, SINFRUG00000153704 |  |
| 29 | SINFRUG00000149811, SINFRUG00000149810 |  |
| 30 | SINFRUG00000141675, SINFRUG00000141677 |  |
| 31 | SINFRUG00000150912, SINFRUG00000150911 |  |
| 32 | SINFRUG00000151420, SINFRUG00000151421 |  |
| 33 | SINFRUG00000162634, SINFRUG00000144023 |  |
| 34 | SINFRUG00000165146, SINFRUG00000125310 |  |
| 35 | SINFRUG00000138881, SINFRUG00000138893 |  |
| 36 | SINFRUG00000152530, SINFRUG00000134260 |  |
| 37 | SINFRUG00000139350, SINFRUG00000159661 |  |
| 38 | SINFRUG00000141746, SINFRUG00000141734 |  |
| 39 | SINFRUG00000131238, SINFRUG00000131239 |  |
| 40 | SINFRUG00000121366, SINFRUG00000121364 | *TR-beta* |
| 41 | SINFRUG00000142300, SINFRUG00000142296 |  |
| 42 | SINFRUG00000152430, SINFRUG00000152431 |  |
| 43 | SINFRUG00000126184, SINFRUG00000126189 |  |
| 44 | SINFRUG00000164515, SINFRUG00000154883 |  |
| 45 | SINFRUG00000160508, SINFRUG00000146667 |  |
| 46 | SINFRUG00000120390, SINFRUG00000142860 |  |
| 47 | SINFRUG00000127155, SINFRUG00000127156 |  |
| 48 | SINFRUG00000131034, SINFRUG00000165148 |  |
| 49 | SINFRUG00000139715, SINFRUG00000139713 |  |
| 50 | SINFRUG00000156189, SINFRUG00000121570 |  |
| 51 | SINFRUG00000148808, SINFRUG00000165659 |  |
| 52 | SINFRUG00000140782, SINFRUG00000140781 |  |
| 53 | SINFRUG00000161472, SINFRUG00000135016 |  |
| 54 | SINFRUG00000151388, SINFRUG00000151387 |  |
| 55 | SINFRUG00000162484, SINFRUG00000124671 | *Fash1* |
| 56 | SINFRUG00000125603, SINFRUG00000160800 |  |
| 57 | SINFRUG00000130361, SINFRUG00000130362 |  |
| 58 | SINFRUG00000160937, SINFRUG00000147597 |  |
| 59 | SINFRUG00000127695, SINFRUG00000128884 |  |
| 60 | SINFRUG00000153677, SINFRUG00000153673 |  |
| 61 | SINFRUG00000161097, SINFRUG00000136281 |  |
| 62 | SINFRUG00000141312, SINFRUG00000141311 |  |
| 63 | SINFRUG00000141198, SINFRUG00000145223 |  |
| 64 | SINFRUG00000161666, SINFRUG00000129874 |  |
| 65 | SINFRUG00000153380, SINFRUG00000148666 |  |
| 66 | SINFRUG00000165510, SINFRUG00000120908 |  |
| 67 | SINFRUG00000164292, SINFRUG00000163449 |  |
| 68 | SINFRUG00000141943, SINFRUG00000141940 |  |
| 69 | SINFRUG00000123564, SINFRUG00000123565 |  |
| 70 | SINFRUG00000126702, SINFRUG00000153244 |  |
| 71 | SINFRUG00000144925, SINFRUG00000144915 |  |
| 72 | SINFRUG00000162084, SINFRUG00000165245 |  |
| 73 | SINFRUG00000143787, SINFRUG00000143786 | *c-FosLb* |
| 74 | SINFRUG00000138736, SINFRUG00000125829 |  |
| 75 | SINFRUG00000149914, SINFRUG00000163840 |  |
| 76 | SINFRUG00000152306, SINFRUG00000152292 |  |
| 77 | SINFRUG00000160274, SINFRUG00000140267 |  |
| 78 | SINFRUG00000160542, SINFRUG00000162003 | *SLP-1* |
| 79 | SINFRUG00000161394, SINFRUG00000163068 |  |
| 80 | SINFRUG00000134492, SINFRUG00000133305 | *Sox17* |
| 81 | SINFRUG00000133135, SINFRUG00000133137 | *nkx2.9, pax9* |
| 82 | SINFRUG00000148673, SINFRUG00000148676 |  |
| 83 | SINFRUG00000137297, SINFRUG00000161299 |  |
| 84 | SINFRUG00000126443, SINFRUG00000131220 |  |
| 85 | SINFRUG00000123105, SINFRUG00000123103 |  |
| 86 | SINFRUG00000131813, SINFRUG00000136721 |  |
| 87 | SINFRUG00000124439, SINFRUG00000124437 |  |
| 88 | SINFRUG00000160916, SINFRUG00000162597 |  |
| 89 | SINFRUG00000160456, SINFRUG00000156588 |  |
| 90 | SINFRUG00000138842, SINFRUG00000138835 |  |
| 91 | SINFRUG00000124021, SINFRUG00000161393 |  |
| 92 | SINFRUG00000127266, SINFRUG00000127264 |  |
| 93 | SINFRUG00000144564, SINFRUG00000144566 |  |
| 94 | SINFRUG00000151239, SINFRUG00000151240 |  |
