# Supplementary material for: TFCONES: A database of vertebrate transcription factor-encoding genes and their associated conserved noncoding elements
Source: BMC Genomics. 2007 Nov 29;8:441. doi: 10.1186/1471-2164-8-441 (PMC2148067; doi:10.1186/1471-2164-8-441)
Supplement: Additional data file 5 — Conserved clusters of human, mouse and fugu TF-encoding genes. An asterisk indicates a human TF-encoding gene that has no ortholog in fugu but is located in a Hox conserved syntenic block. [file 1471-2164-8-441-S5.doc]

Additional data file 5. Conserved clusters of human, mouse and fugu TF-encoding genes. An asterisk indicates a human TF-encoding gene that has no ortholog in fugu but is located in a *Hox* conserved syntenic block.

| **No.** | **Number of genes in cluster** | **Gene IDs (human)** | **Gene names** | **Number of CNEs** | **Total length of CNEs (bp)** |
| --- | --- | --- | --- | --- | --- |
|  | 12 | ENSG00000105991, ENSG00000105996, ENSG00000105997, ENSG00000197576, ENSG00000106004, ENSG00000106006*, ENSG00000122592*, ENSG00000078399, ENSG00000153807, ENSG00000005073, ENSG00000106031, ENSG00000106038 | *HOXA1, HOXA2, HOXA3, HOXA4, HOXA5, HOXA6*, HOXA7*, HOXA9, HOXA10, HOXA11, HOXA13, EVX1* | 25 | 3,011 |
|  | 10 | ENSG00000120094, ENSG00000173917, ENSG00000120093, ENSG00000182742, ENSG00000120075, ENSG00000108511, ENSG00000120087*, ENSG00000120068, ENSG00000170689, ENSG00000159184 | *HOXB1, HOXB2, HOXB3, HOXB4, HOXB5, HOXB6, HOXB7*, HOXB8, HOXB9, HOXB13* | 21 | 2,333 |
|  | 10 | ENSG00000174279, ENSG00000128714*, ENSG00000170178, ENSG00000128713, ENSG00000128710, ENSG00000128709, ENSG00000175879*, ENSG00000170166, ENSG00000128652, ENSG00000128645* | *EVX2, HOXD13*, HOXD12, HOXD11, HOXD10, HOXD9, HOXD8*, HOXD4, HOXD3, HOXD1** | 12 | 2,344 |
|  | 9 | ENSG00000123364, ENSG00000123407, ENSG00000123388, ENSG00000180818, ENSG00000180806, ENSG00000037965, ENSG00000197757, ENSG00000172789, ENSG00000198353 | *HOXC13, HOXC12, HOXC11, HOXC10, HOXC9, HOXC8, HOXC6, HOXC5, HOXC4* | 19 | 2,234 |
|  | 3 | ENSG00000137090, ENSG00000064218, ENSG00000173253 | *DMRT1, DMRT3, DMRT2* | 7 | 1,445 |
|  | 2 | ENSG00000126778, ENSG00000100625 | *SIX1, SIX4* | 5 | 402 |
|  | 2 | ENSG00000176842, ENSG00000159387 | *IRX5, IRX6* | 81 | 13,070 |
|  | 2 | ENSG00000187098, ENSG00000114861 | *MITF, FOXP1* | 47 | 8,099 |
|  | 2 | ENSG00000174963, ENSG00000152977 | *ZIC4, ZIC1* | 28 | 3,642 |
|  | 2 | ENSG00000008197, ENSG00000008196 | *TFAP2D, TFAP2B* | 10 | 1,224 |
|  | 2 | ENSG00000188620, ENSG00000188816 | *XP_291716.5, NP_005510.1* | 10 | 2,072 |
|  | 2 | ENSG00000134954, ENSG00000151702 | *ETS1, FLI1* | 6 | 652 |
|  | 2 | ENSG00000144355, ENSG00000115844 | *DLX1, DLX2* | 5 | 1,006 |
|  | 2 | ENSG00000111046, ENSG00000111049 | *MYF6, MYF5* | 1 | 94 |
|  | 2 | ENSG00000135457, ENSG00000184271 | *TFCP2, POU6F1* | 1 | 56 |
|  | 2 | ENSG00000162761, ENSG00000143171 | *LMX1A, RXRG* | 1 | 78 |
|  | 2 | ENSG00000175832, ENSG00000005102 | *ETV4, MEOX1* | 1 | 75 |
|  | 2 | ENSG00000108813, ENSG00000064195 | *DLX4, DLX3* | 0 | 0 |
