# Supplementary material for: TFCONES: A database of vertebrate transcription factor-encoding genes and their associated conserved noncoding elements
Source: BMC Genomics. 2007 Nov 29;8:441. doi: 10.1186/1471-2164-8-441 (PMC2148067; doi:10.1186/1471-2164-8-441)
Supplement: Additional data file 6 — Distribution of lengths of (A) human-mouse and (B) human-fugu CNEs. [file 1471-2164-8-441-S6.doc]

Additional data file 6. Distribution of lengths of (A) human-mouse and (B) human-fugu CNEs.

**A**

**B**
