# Supplementary material for: TFCONES: A database of vertebrate transcription factor-encoding genes and their associated conserved noncoding elements
Source: BMC Genomics. 2007 Nov 29;8:441. doi: 10.1186/1471-2164-8-441 (PMC2148067; doi:10.1186/1471-2164-8-441)
Supplement: Additional data file 7 — Top twenty TF-encoding genes associated with the highest density of human-fugu CNEs. For genes that are part of conserved clusters, we averaged out the number or length of CNEs present in the whole cluster over the number of genes in that cluster. CNE density is defined as the number of bases located in CNEs per 100 bp of non-repetitive noncoding sequence in the longest orthologous fugu gene locus. [file 1471-2164-8-441-S7.doc]

Additional data file 7. Top twenty TF-encoding genes associated with the highest density of human-fugu CNEs. For genes that are part of conserved clusters, we averaged out the number or length of CNEs present in the whole cluster over the number of genes in that cluster. CNE density is defined here as the number of bases located in CNEs per 100 bp of non-repetitive noncoding sequence in the longest orthologous fugu gene locus.

| **Gene ID** | **Gene name** | **Description** | **CNE bases per 100 bp of fugu sequence** |
| --- | --- | --- | --- |
| ENSG00000170561 | *IRX2* | Iroquois-class homeodomain protein IRX-2 | 67.27 |
| ENSG00000178860 | *MSC* | Musculin | 28.97 |
| ENSG00000139515 | *IPF1* | Insulin promoter factor 1 | 28.27 |
| ENSG00000114861 | *FOXP1* | Forkhead box protein P1. | 22.07 |
| ENSG00000187098 | *MITF* | Microphthalmia-associated transcription factor. | 22.07 |
| ENSG00000108270 | *AATF* | Protein AATF | 15.80 |
| ENSG00000105880 | *DLX5* | Homeobox protein DLX-5. | 14.16 |
| ENSG00000109132 | *PHOX2B* | Paired mesoderm homeobox protein 2B | 9.67 |
| ENSG00000007372 | *PAX6* | Paired box protein Pax-6 | 9.47 |
| ENSG00000164853 | *XP_496843.1* | PREDICTED: similar to Uncx4.1 | 9.20 |
| ENSG00000128573 | *FOXP2* | Forkhead box protein P2 | 8.79 |
| ENSG00000153234 | *NR4A2* | Orphan nuclear receptor NR4A2 | 8.34 |
| ENSG00000181449 | *SOX2* | Transcription factor SOX-2. | 7.98 |
| ENSG00000130675 | *HLXB9* | Homeobox protein HB9. | 7.64 |
| ENSG00000169946 | *ZFPM2* | Zinc finger protein ZFPM2 | 7.61 |
| ENSG00000139800 | *ZIC5* | Zinc finger protein ZIC 5 | 7.47 |
| ENSG00000170549 | *IRX1* | Iroquois-class homeodomain protein IRX-1 | 7.45 |
| ENSG00000006377 | *DLX6* | Homeobox protein DLX-6. | 7.24 |
| ENSG00000032514 | *ERCC6* | DNA excision repair protein ERCC-6 | 7.06 |
| ENSG00000134138 | *MEIS2* | Homeobox protein Meis2 | 6.96 |
