# Supplementary material for: TFCONES: A database of vertebrate transcription factor-encoding genes and their associated conserved noncoding elements
Source: BMC Genomics. 2007 Nov 29;8:441. doi: 10.1186/1471-2164-8-441 (PMC2148067; doi:10.1186/1471-2164-8-441)
Supplement: Additional data file 8 — Significantly over-represented and under-represented Gene Ontology terms (P < 0.01) of CNE-associated human TF-encoding genes. Group A denotes 385 of 389 CNE-associated TF-encoding genes with Gene Ontology annotation, while Group B denotes 804 of 816 orthologous TF-encoding genes with Gene Ontology annotation. P-values marked with a negative sign denote significant depletion. The analysis was carried out using GOstat (Beissbarth and Speed 2004). [file 1471-2164-8-441-S8.doc]

Additional data file 8. Significantly over-represented and under-represented Gene Ontology terms (*P* < 0.01) of CNE-associated human TF-encoding genes. Group A denotes 385 of 389 CNE-associated TF-encoding genes with Gene Ontology annotation, while Group B denotes 804 of 816 orthologous TF-encoding genes with Gene Ontology annotation. *P*-values marked with a negative sign denote significant depletion. The analysis was carried out using GOstat (Beissbarth and Speed 2004).

| **GO ID** | **GO description** | **Group A (385)** | **Group B (804)** | ***P*-value** |
| --- | --- | --- | --- | --- |
| GO:0007275 | multicellular organismal development | 211 | 347 | 2.22 × 10^-7^ |
| GO:0032501 | multicellular organismal process | 216 | 361 | 6.08 × 10^-7^ |
| GO:0007399 | nervous system development | 87 | 120 | 2.47 × 10^-6^ |
| GO:0032502 | developmental process | 231 | 398 | 3.67 × 10^-6^ |
| GO:0007420 | brain development | 39 | 46 | 8.08 × 10^-5^ |
| GO:0007417 | central nervous system development | 49 | 62 | 8.08 × 10^-5^ |
| GO:0048731 | system development | 161 | 266 | 8.08 × 10^-5^ |
| GO:0048856 | anatomical structure development | 176 | 296 | 8.19 × 10^-5^ |
| GO:0007389 | pattern specification process | 34 | 43 | 0.0036 |
| GO:0048513 | organ development | 128 | 214 | 0.00386 |
| GO:0043412 | biopolymer modification | 4 | 33 | -0.00375 |
| GO:0019538 | protein metabolic process | 9 | 50 | -0.00186 |
| GO:0044267 | cellular protein metabolic process | 8 | 48 | -0.00134 |
| GO:0044260 | cellular macromolecule metabolic process | 8 | 48 | -0.00134 |
